# Construction Notes

**Published:** 1907-02-16

**Authors:** 


### CONSTRUCTION NOTES.

### THE RADCLIFFE INFIRMARY.

The reorganisation, reconstructon, and improvement of this hospital, now completed, include the kitchen and the heating apparatus from the central boiler for the operating theatre and the whole of the centre block. The balconies for the outdoor treatment of patients have also been completed, and an $x$-ray apparatus has been acquired and is now in full working order.

### HARROGATE ROYAL BATH HOSPITAL.

During the past year a new drainage system has been put down at a cost of £715. Certain additions are, however, not quite complete, and will entail a further expenditure of £180. The whole of the old drains have been taken up, and the new pipes have been laid down in six-inch concrete casing and have been thoroughly tested. The site of the hospital is on reclaimed moorland, on very shifty, peaty subsoil, and the renovation of the drainage system was attended with great difficulties.

### BARRED WINDOWS IN POOR-LAW HOSPITALS.

The Visiting Committee of the Union Hospital at Hope, Salford, recommended that the lavatory windows on the third and second floors should be barred, as a precautionary measure. The recommendation was opposed by a Guardian on the ground that blocks could be so placed as to prevent the window from being opened to a dangerous point. This was again objected to on the ground that it would impede ventilation. To bar up the windows would entail an expenditure of £200, while the blocks would be much cheaper.

### THE BRISTOL GENERAL HOSPITAL.

The new isolation wards of the Bristol General Hospital have just been opened. They comprise an entirely new block, built from plans by Messrs. Oatley and Lawrence. The new block is on the east side of the entrance roadway, and consists of two floors. On each floor there are two wards and one duty room, besides a private, single-bedded ward. The building thus accommodates ten patients. In addition there are nursing quarters, including sitting-rooms. The sanitary arrangements are on an improved plan. A feature of the new wing are the Gilmour patent doors made of wood sections without panels or sinkings, and therefore uniformly smooth. All the woodwork as well as the walls are to be enamelled in white, and the flooring is of the jointless, polished "Eubeolith" pattern. The building is warmed on the low pressure water system, and ventilated by several electric fans. The estimated cost is £11,000, and about £3,000 has been received or promised.

### THE WARRINGTON INFIRMARY.

During the last decade Warrington has almost doubled its population, and the local infirmary has become far too small. A sum of £11,000 has been raised towards the £15,000 required for its reconstruction. With this sum in hand the Board has decided to commence operations, and the foundation-stone of the new block was laid last week by Lord Lilford. The scheme for the new buildings provides for the erection of a pavilion and nurses' home. The pavilion, which is to be the main extension to the infirmary, is to be three stories high, and will give 66 additional beds. The aspect of the pavilion is due north and south, so that patients will have most of the sunshine on fine days. Each floor is to consist of a single ward, accommodating 22 patients. There will be the usual sanitary additions, and balconies will be provided for the convenience of convalescents. Each ward will have its own kitchen on the same floor, and ample provision has been made for fire exits in the shape of a large fire-escape staircase, to which easy access is obtained from the wards. The building is to be heated by means of a double arrangement of hot-water pipes and open faience stoves with descending flues which will add much to the comfort of the wards. The architects are Messrs. William and Segar Owen, and the contract has been given to Mr. Davenport, of Stockton Heath.

